# Exploring Early Childhood Educators’ Perceptions and Practices Towards Gender Differences in STEM Play: A Multiple-Case Study in China

**DOI:** 10.1007/s10643-023-01499-3

**Published:** 2023-05-19

**Authors:** Simeng Wang

**Affiliations:** Faculty of Education, Monash University, Melbourne, Victoria Australia

**Keywords:** Early childhood education, STEM, Gender-stereotype, Chinese early childhood educator, Gender-inclusive educational setting

## Abstract

Various gender biases have been demonstrated in early childhood educators (ECEs) with unintentional preferential treatment provided to boys during STEM-related play activities. These biases could impact young girls’ identity formation, resulting in continued underrepresentation of women in STEM domains in future. In China, however, little research has been conducted on how ECEs perceive gender equity of STEM fields. Consequently, this study aims to close this gap by investigating the educators’ perceptions on and responses to gender differences in STEM play, drawing on the cultural-historical theory and incorporating feminist perspectives. Adopting a multiple-case study approach, this study collected perceptions and experiences of six Chinese in-service ECEs regarding STEM play and gender-related issues. The participants recognized and valued children’s equal involvement in STEM play, but failed to preclude ingrained gender preconceptions, leading to contradictory beliefs and performs. Meanwhile, Chinese ECEs considered prejudices from the external environment and peer influence the main obstacles to gender inclusion. Inclusive practices and emphasises are thus discussed relating to ECEs’ multiple roles in supporting gender-neutral environments for STEM play. These preliminary findings shed light on attaining gender equity in STEM within the context of a feminist discourse, and provide Chinese educators, leaders and even the educational system with pioneering information. However, further research on ECEs’ underlying stereotypes and teaching practices is still warranted to examine future professional development possibilities, support ECEs in reducing obstacles to girls’ STEM engagement, and ultimately create a welcoming and inclusive STEM play space for girls.

## Introduction

### STEM and Gender Issues

STEM education (the acronym of science, technology, engineering and mathematics), as an emerging interdisciplinary concept, influences children’s future choices of college majors and careers, but the gender difference in STEM domains has become a serious and persistent issue globally (Fitzgerald & Corrigan, 2021). In higher education, only 30% of women around the globe pursue STEM-related fields, and they are less likely than men to choose STEM careers (Chavatzia, 2017; Department of Industry, Innovation & Science, 2019). In light of the significance of STEM innovation in the twenty-first century, it is both detrimental to their personal development societal equity for women to be absent from STEM fields (Master & Meltzoff, 2017).

Rogers et al. (2021) ascribe gender inequalities in STEM to early childhood gendered socialization, which influences the development of children's academic identities and interests. Nonetheless, the effects of gender biases in STEM on younger children are seldom investigated (Tippett & Milford, 2017).

Early childhood (EC) is a vital period for establishing and developing gender roles and STEM identities (Campbell et al., 2020). EC instructors play a significant role in this process since their stereotyped rhetoric and unintended favouritism toward boys in STEM participation may prevent girls from participating in STEM activities and developing STEM-related abilities (Hand et al., 2017; Martin et al., 2018; Morgan et al., 2016). According to the ecological model of Bronfenbrenner, educators’ gender identities and gender-biased teaching techniques may directly influence children’s gender-isolated behaviours, interacting in the microsystem of children (Bronfenbrenner & Morris, 2006). To investigate how gender biases adversely affect girls' STEM engagement, an in-depth investigation of early childhood educators’ (ECEs) perceptions and behaviours is valuable and necessitated. It might provide some theoretical and applied guidance for ECEs to reduce gender stereotyping in the EC educational environment, hence contributing to a more gender-balanced distribution in STEM-related workplaces.

### STEM in the Chinese Context

Historically, China followed stereotypical gender roles on labour allocations, influenced by the conventional Confucian heritage of “men outside and women inside the home” (Yang & Gao, 2019, p. 1716). As a result, only 7.38% of the academicians in the Chinese Academy of Engineering (2021) and Chinese Academy of Sciences (2021) elected in 2021 were women, signifying a lack of female representation in STEM occupations.

As China has grown rapidly in science and technology over the past few decades, there is still considerable scope to examine and develop STEM careers and education (Gao, 2018; Tao, 2019). Moreover, STEM as an emerging educational technique in China is rarely explored in contemporary research, in contrast to western nations, which have an abundance of pertinent studies (Li et al., 2019). Therefore, research on gender biases in STEM could contribute to enlarging China’s talent pool in science and technology. As a result of the many similarities between countries (Dong et al., 2020), it may also offer insights into gender equity in STEM industries in Asia and the rest of the world (Ayres, 2016; Holmlund et al., 2018), especially in the teaching practices of educators who are used to traditional pedagogical methods.

### The Current Study

A review of the contemporary literature on Chinese EC education revealed two noteworthy research gaps. A majority of the literature examines STEM teaching approaches and enhancing educators’ confidence in STEM integration (Dong et al., 2020; Li & Chen, 2016; Tao, 2019), but few empirical studies examine gender issues in STEM education for young children. Furthermore, play has been undervalued as children’s primary learning environment in China, impacted by the Confucian heritage that emphasizes academic training over play, to support students to achieve fame, wealth and status in the Imperial Civil Examination (Cheng, 2011). In early childhood education, such an exam-based tradition discourages play since it distracts children from rigorous learning, which could be reflected in the Confucian classics *Three-Character Classics* (三字经), claiming *“Diligence has merit, but the play has no benefit”* (勤有功, 戏无益) (Wang, 1223–1296). Under this cultural ethos, play is seen as the antithesis of formal education, especially in early childhood (Lin et al., 2018). Accordingly, there has been little study into using play as a vehicle to increase children's STEM learning outcomes and abilities.

Meanwhile, the Chinese government has recently acknowledged both the importance of gender equity and the role of play in early childhood education. The Chinese government has vigorously promoted the *National Rejuvenation through Science and Technology* strategy over the last two decades to promote equitable access to science-related courses, especially for girls, the gender group who was severely undervalued in STEM (Liu, 2018; Zhu, 1995). Furthermore, the newly published *Guideline for Assessing the Quality of Kindergarten Care and Education of China* (Ministry of Education of the People’s Republic of China, 2022) and *14th Five-Year Action Plan in Early Years Education Development* (Ministry of Education of the People’s Republic of China, 2021) both place a high value on integrated learning, which incorporates STEM concepts into play-based activities, safeguarding children’s personalized needs of learning development in rich and insightful play environments.

This research is significant in elevating Chinese ECEs’ consciousness of gender equality in early childhood STEM education, as well as providing chances for ECEs to share their stories, including personal experiences, sentiments, and opinions about gender inequalities in STEM play. Therefore, the following questions will be addressed in this study:

**RQ1:** What are Chinese ECEs’ perceptions about gender differences in STEM play?

**RQ2**: What do Chinese ECEs see as obstacles to girls’ engagement in STEM play?

**RQ3**: What are Chinese ECEs’ practices to empower girls in STEM play?

## Literature and Conceptual Framing

### Gender Differences in STEM Play

Boys and girls have been shown to have different purposes of using STEM-related skills in play. Gold (2014) noticed that in technological plays boys’ interest in constructing is activity oriented. They tend to view technological constructions, such as breaking and reconstructing buildings, as their centre of play (Hallström et al., 2014). Girls, however, prefer to apply technological skills as an aid to achieve their social purposes, such as building a fence for animals in their dramatic narrative play (Hallström et al., 2014).

Furthermore, researchers notice distinct gender biases in EC play areas. Boys usually act as dominators by quickly occupying construction areas, whereas girls have difficulty accessing these areas with equal opportunities to participate (Fleer, 2019). Interestingly, when girls “return” to home corner areas, they could recover a sense of authority (Fleer, 2019). Meanwhile, girls in engineering play are more likely to be bystanders and helpers for boys rather than creating their own block works (Hallström et al., 2014; Stephenson et al., 2021a). These studies reveal that different play areas empower boys and girls with distinct rights and senses of belonging. Girls’ lack of a sense of belonging in STEM play spaces thus signifies the future barriers to entry and share ownership of STEM fields (Master & Meltzoff, 2017).

Additionally, girls might be less confident in participating in STEM activities than boys. From the observation of Hallström et al. (2014), boys are very active in expressing ideas verbally and physically in simulated science experiments, while girls spontaneously distance themselves from experimental areas. Girls’ behaviours in STEM play might be triggered by the cultural pressure and traditional gender roles, implying girls are not a match for STEM (Fleer, 2019). These stereotypes could lead to girls’ lacking academic preparation and interest in STEM engagement, which will be examined further in the next section,* Barriers to Girls' Engagement in STEM*.

### Barriers to Girls’ Engagement in STEM

Researchers attribute girls’ low STEM engagement to gender stereotypes that deem boys have higher competence to resolve STEM problems (Campbell et al., 2020; Master & Meltzoff, 2017). Due to the close interrelationship between children and their surrounding social context (Vygotsky, 1978), the subtle but persistent hostility from different layers of society towards girls’ STEM experiences may cumulatively cultivate girls’ lower STEM identities and interests, thereby undermining girls’ equal access to STEM fields (Fleer, 2019; Stephenson et al., 2021a).

From macroscopical perspectives, children’s STEM engagement could be impacted by mainstream ideologies and policies in EC settings. A Turkish study conducted by Çetin et al. (2020) indicates that children in EC settings are showing increasingly evident gender differences in STEM play over time, since they are exposed to the joint influence of structured education in formal EC institutions and misinformation from social ideology. Even in Sweden, one of the most gender-equitable countries, educators are influenced by preconceptions on children’s STEM development, believing that gender differences are innate (Gullberg et al., 2017).

Furthermore, children’s STEM awareness and play preferences are influenced by the gendered messages conveyed by media and toy marketing. Okanda and Taniguchi (2021) declare that Japanese cartoons, such as *Astro Boy* and *Doraemon*, usually depict robots as boys, implying robots signify masculinity. Moreover, the Institution of Engineering and Technology (2016) in the UK found that on search engines and retailer advertising, commercials of STEM toys targeting boys are three times more than those targeting girls. Even LEGO, which claims to balance the play preferences of boys and girls, still portrays children’s biased social roles and possibly contribute to restricting girls’ interests in STEM development (Reich et al., 2017).

Parents with preconceived attitudes towards children’s STEM competence also considerably discourage girls’ STEM participation. Research demonstrates that parents with more positive attitudes towards STEM could develop girls’ choices on typically male-dominated STEM careers (Mulvey & Irvin, 2018). However, more studies uncover that parents are prone to expressing higher expectations, raising more advanced questions, and paying more attention to boys in STEM-related activities, which could increase the gap between boys and girls in STEM attitudes and engagement (Coyle & Liben, 2018).

### The Educator as a Critical Influence in STEM Play

Many educators recognise the necessity of breaking the traditional girls’ STEM role and intentionally challenging gender stereotypes (Gullberg et al., 2017; Hallström et al., 2014; Stephenson et al., 2021a). Some educators redefine their roles as co-participants in STEM play to eliminate gender barriers through immersing themselves in imaginative play without a specific gender (Hallström et al., 2014; Stephenson et al., 2021a). Others endeavour to cultivate non-differentiated play environments by providing gender-equitable play resources and encouraging boys and girls to regularly exchange play areas (Chapman, 2016; Lynch, 2015). Still some educators actively shift their STEM teaching from teacher-organised to child-led, enabling children, especially girls, to comfortably challenge new ideas in free-controlled play without fear of making mistakes (Rushton & King, 2020).

Nevertheless, verbally claiming to set the same expectations for boys’ and girls’ STEM activities does not mean that educators are actually free from gender stereotypes in practice. Various studies have unveiled unconscious gender bias in teaching. Studies from Turkey (Çetin et al., 2020), China (Chen & Rao, 2011) and Sweden (Gullberg et al., 2017) all reveal that ECEs’ preconceptions about children’s interests often limit children’s play choices by gender-labelling play resources which are otherwise neutral, differentiating boys and girls in separate groups and assigning them different activities based on genders. Likewise, three Australian studies demonstrate that ECEs usually pay more attention to developing boys’ capabilities in “masculine” areas such as architecture and engineering; they ask boys more open questions, set different expectations for boys and girls, and name children with gender-related terms, such as engineers and female engineers (Fleer, 2019; Stephenson et al., 2021a, 2021b). These practices unintentionally convey to girls that STEM is a boy-dominated field, imposing unjustified negative impacts on girls’ future STEM development. These studies seem to reveal the conflicting beliefs and practices of EC educators, suggesting educators’ “consciousness regarding girls’ engagement is in its early stages of development” (Stephenson et al., 2021b, p. 8).

### Approaches to Develop Girls’ STEM Engagement

Current literature suggests that strategies dedicated to promoting gender inclusion in STEM play could be broadly grouped into two categories: simulating social-realistic scenarios with imaginary play (Gold et al., 2015; Stephenson et al., 2021a, 2021b) and providing free-choice play resources (He, 2018; Gullberg et al., 2017; Rushton & King, 2020).

According to Vygotsky (1967), children could reshape their social behaviours in a collective imaginary situation when actively interacting in “as if” scenarios. The *Conceptual Playworld* is a worthy example of collective imagination, referring to children’s changing social situation when entering a *Playworld* created by educators without explicit gender connotations (Stephenson et al., 2021a). In this gender-neutral “world”, girls can break out of the traditional discourse of STEM and dominate the play area where they did not have the opportunity to participate (Stephenson et al., 2021b). Likewise, Gold et al. (2015) also adheres to Vygotsky’s theory by indicating that dramatic play areas support children’s social ideas through communication, interpretation and coordination. These valuable competencies provide the context and potential for developing children’s STEM thinking model.

Free-choice play is considered as another effective strategy that promotes children’s equal STEM participation. Rushton and King (2020) indicate that *Making activities* can encourage children to apply technological materials to develop hands-on skills in designing and building, providing them with free choice to equally engage in STEM activities. Coincidentally, Chinese *Anji play* presents a similar concept by providing children with large yards as free-play spaces and encouraging them to creatively play with local materials such as wood bricks, ladders and tyres, without interference from ECEs (He, 2018). While no current study from China has explicitly discussed the benefits of gender inclusion, *Anji play* as a breakthrough from the Chinese traditional teacher-led settings, has the potential to empower young children with equal rights to participate in STEM activities. Such a philosophy of reducing educators’ intervention in free-choice play is also introduced as *Essentia discourse* in Swedish kindergartens to develop children’s STEM initiatives (Gullberg et al., 2017). Immersing in these open playful environments, boys or girls could both explore science and engineering topics in their comfortable and confident ways, with which they previously had limited experience.

### Conceptual Framework: Social Contexts Overlapping CHT and Feminist Discourse

This research employs Vygotsky’s (1997) cultural-historical theory (CHT) under the philosophical worldview of constructivism to explore educators’ different beliefs and everyday practices that significantly influence children’s development. On the one hand, educators’ beliefs could directly influence children’s development during their interactions, since children’s learning relies significantly on everyday social settings that they are exposed to (Ridgway et al., 2015). On the other hand, CHT emphasises the dynamics between routine activities, social norms and discourses by declaring children’s development is a continuous and evolving process (Hedegaard, 2009; Ødegaard & Krüger, 2012).

**Fig. 1:**
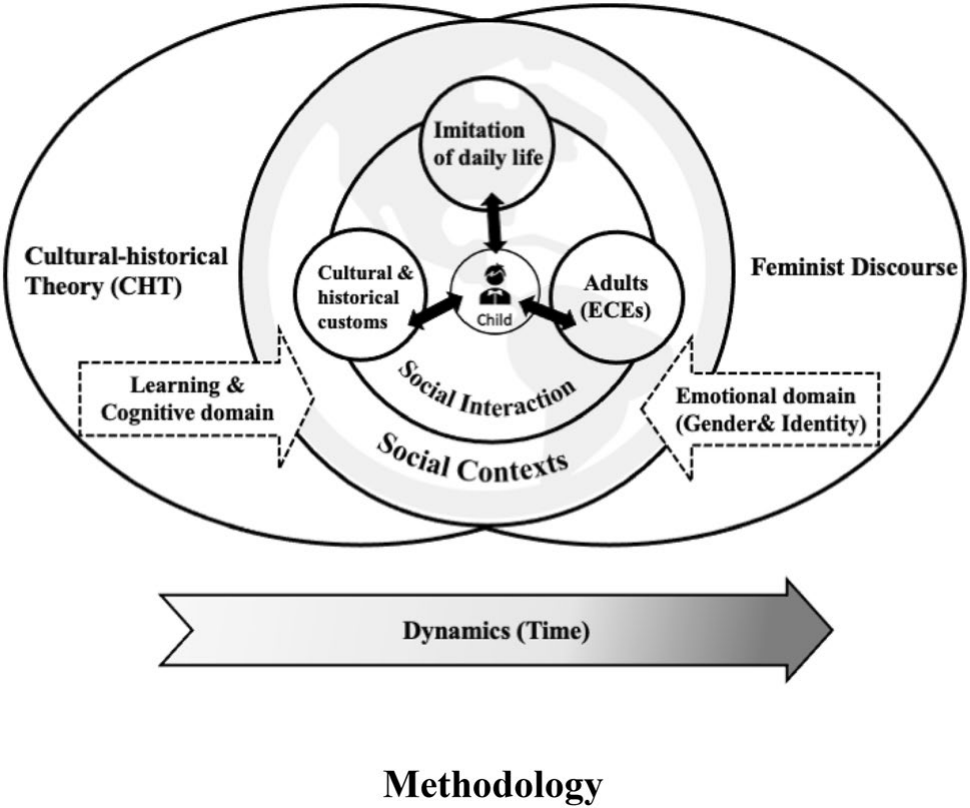
The conceptual framework combining CHT and Feminist discourse

Meanwhile, a feminist discourse as another critical analytical toolkit to explore the equity of STEM fields, will complement the cultural-historical framework to investigate educators’ gender bias in relation to STEM activities (Heybach & Pickup, 2017). CHT places a stronger emphasis on children’s development from learning and cognitive domains in social interaction, while feminist theory focuses on gender and identity in the emotional domain (John-Steiner, 1999). Consequently, rationally utilising the complementarity of theories rather than their opposition could allow a more holistic vision for exploring the current research questions. While the cultural-historical standpoint aims to support educators to reflect on and overcome common and preconceived misconceptions in practice, it lacks consideration of children’s learning development from a gender perspective. Hence, a feminist perspective could be applied to complement this absent focus by investigating ECEs’ understanding and actions on the privileged issue of gender identity in STEM. The combination of perspectives thus provides a deeper insight into whether or how ECEs can transform gender-stereotypical discourses and practices to cultivate inclusive learning and playing environments. The design framework of this research is in line with John-Steiner’s (1999) belief that progress in constructing a more comprehensive cultural-historical theory will be made relying upon feminist works. Figure 1 below is thus composed to illustrate the interactive and complementary use of the two theoretical perspectives in this research.

## Methodology

### A Multiple-Case Study

This research employs a case study approach, investigating educators’ thoughts and practices in a particular education system (China) regarding gender differences in specific settings (STEM play) with a specific age group (early childhood) (Baxter & Jack, 2008). As the intended participants are more than one ECE, a multiple-case study was applied by regarding each Chinese ECE as a separate case. Since participants come from various educational institutions, with different levels of education training background and years of teaching, their distinctive perceptions and approaches on this research topic are well-worth exploring and examining. Under such circumstances, a multiple-case study could support the research in comprehending the uniqueness and complexity of each case by constructing a mutual platform that allows for discovering and analysing similarities as well as differences between these cases across settings (Baxter & Jack, 2008; Stake, 2006).

### Participant Recruitment

Participants were recruited from two popular Chinese social media: *Xiaohongshu* and *WeChat*, by publishing an invitation advertisement. Applying the maximum variation sampling, the researcher selected six ECEs to participate from 11 potential participants who expressed their interests in this program based on different profiles. Maximising the range of characteristics of the sample could enhance the potential for generalization by involving broader types of participants with a small but representative sample, including those employed in diverse educational systems, with varied educational backgrounds and years of teaching experience (Gray, 2003). The six ECEs were invited to voluntarily participate in this research after reading the explanatory statement and signing the consent form. The demographic details of the participants are displayed in Table 1 below.

**Table 1:** Participant demographics

Participant	Gender	Teaching years	Education system	Teaching age group	Province
Sarah	Female	1 year	Private	2–3	Sichuan
Emily	Female	21 years	International	3–4	Beijing
Amber	Female	4 years	Public	3–4	Hainan
Rennie	Female	0.5 year	Public	4–5	Hunan
May	Female	16 years	Public	4–5	Liaoning
Cathy	Female	10 years	Private	5–6	Liaoning

Note: the names of participants are pseudonyms

### Data Collection

Data were collected through an individual semi-structured interview with each participant. Such a highly reciprocal data collection approach, which empowers participants equal opportunities to freely express their perceptions, is deemed most suitable for this project and fits into the conceptual framework combining CHT and feminist discourses (Bryman, 2016). Open-ended questions were asked, including ECEs’ inherent beliefs and experiences, obstacles and practices to promote gender equity in STEM play. Based on participants’ responses, the researcher extended the pre-designed questions and flexibly asked additional questions for further discussion (Bold, 2012).

To avoid potential health risks from the COVID-19 Pandemic, this research utilized online interviews through *Tencent Meeting* and *WeChat Calling*, at participants’ preference. This online format is more flexible than the traditional face-to-face interview, allowing for the recruitment of participants without geographical restrictions and saving time for both parties from travelling (Bryman, 2016). Moreover, to ensure the accuracy and detailedness of the data, the interviews were conducted in the participants’ first language, Mandarin, to avoid misunderstandings out of language barriers, and recorded the audios with their consent.

To compensate for the limitations of online interviews, which disallowed in-person observation of the ECEs’ work environment, they were encouraged to prepare some photographs at work related to the STEM play environment (without showing the children’s faces), such as toys, resources, and daily activities. These photographs as a supplement to the conversation, stimulated ECEs to critically reflect on their taken-for-granted practices by visually revisiting their familiar environment, and provided the researcher with a diverse means of understanding and capturing their deeper thoughts, thus revealing new foci and issues (Bryman, 2016).

### Data Analysis

The data from interviews in this study were analysed by reflexive thematic analysis (Braun & Clarke, 2019). After converting the interview audio to text, the researcher highlighted and coded all potentially important information through re-reading the whole database, aiming to generate succinct and shorthand labels for snippets of information that might be of relevance to the research question (Byrne, 2021). First, individual case has been analysed to thoroughly understand ECEs’ unique positions and different practices regarding children’s gender differences in STEM play. Then, thematic analysis was used to continuously analyze and compare details and data from each individual case to discover their similarities and differences, leading to further revisions of the coding scheme (King, 2004). Through the reflexive approach, coded labels with similar concepts or characteristics were categorised into 14 initial themes, before redefining and naming eight ultimate themes that aligned more closely to the research, corresponding to each of the three research questions (Byrne, 2021).

## Findings and Discussion

Findings from the interviews were presented, interpreted and discussed in three sections, built around the study’s three research questions stated earlier, to explore how Chinese ECEs perceive, understand and respond to gender differences in STEM play.

### What are Chinese ECEs’ Perceptions About Gender Differences in STEM Play?

#### Theme 1: The Nature-Nurture Debate About Gender Differences in STEM

When discussing the disparity of gender ratio in STEM careers in the Chinese context, the participants presented distinct understandings related to early childhood education. Cathy and May attributed the STEM differences to nature.

Boys naturally have more advanced logical thinking, which leads to their better performance in logic-related subjects, such as STEM. (Cathy)

Such an ideology of “innatism” supports a biological vision, ascribing the gender imbalance in STEM to innate gender differences, including intelligence levels and cognitive capabilities (Sarseke, 2017). The discourse of Cathy and May revealed their inner perceptions that girls are inherently inferior to boys in STEM. When ECEs consider boys’ and girls’ differentiated performance in STEM play as the “natural expression of biological differences”, the need to equate the number of boys and girls in STEM engagement becomes less significant (Browne, 2004, p. 7).

However, this viewpoint was explicitly doubted by the other four participants, who argued that STEM differences are nurtured. Emily, an experienced ECE from a Beijing international kindergarten, believed that children’s STEM abilities are groomed later in life by their families, schools and social environment. Similarly, Sarah, a newer ECE, also supported the role of nurture:

Boys’ and girls’ STEM interests and skills start at the same level as children are ‘blank paper’ waiting to be written on. The differences manifesting later mainly stem from the subtle transmission of traditional perceptions from society, such as ‘girls should pursue soft jobs for better taking care of their families. (Sarah)

Such a viewpoint, that children’s abilities are groomed later influencing by the society, is aligned with the feminist discourse (Blickenstaff, 2005) that deems that environmental factors impact the individual child. From the cultural-historical perspective, children’s STEM-related knowledge is cultivated through interacting with their surroundings and transmitting cultural and historical customs (Bozhovich, 2004). Therefore, girls growing up in a gender-biased environment are prone to lag behind boys in STEM performance over time.

These findings on ECEs’ gender-related ideologies are critical to this study since they reveal some ECEs’ attention to and reflection on how acquired nurture impacts girls’ STEM engagement.

#### Theme 2: Claims of Gender Equity May Not Eliminate Subconscious Bias

When discussing gender-related beliefs held in STEM play, all participants claimed that they treat boys and girls equally. Rather than focusing on gender, educators seem to focus more on children’s personal interests. Emily particularly valued the Montessori education system, which advocates establishing an undifferentiated learning environment by providing rich play resources. She noted that “such an environment would give children freedom of choice to construct their own personalities spontaneously”. Emily’s teaching philosophy, one of downplaying gender separation, coincides with the findings of Chapman (2016) and Pardhan and Pelletier (2017), which indicate that some educators’ neutral attitudes of designing games or play resources for children’s interests in a way of neither discouraging nor reinforcing gender roles.

Rennie, an ECE from a public kindergarten, explained that the improved status of women in China has inspired her vision for promoting gender equity in the STEM environment:

Women’s social status in Hunan [the province where she works and lives] is rapidly developing, translating into powerful feminist consciousness to defy deep-rooted gender biases. We ECEs are thus socially responsible to reinforce girls’ values in our teaching practices, by encouraging girls’ active participation in STEM-relevant activities, which were commonly considered boys’ domain. (Rennie)

In line with this study’s conceptual framework of feminist discourse, Rennie voiced female ECEs’ intention to defy the gender-biased paradigm and cultivate gender-equal environments for the current generation to understand STEM (Heybach & Pickup, 2017).

However, as the interviews progressed, and with reflection and introspection, some participants acknowledged their unconscious stereotypes. Amber admitted:

I have always believed that I am impartial to boys and girls, but in retrospect, I may have this subconscious gender bias in my teaching. For example, I thought it was normal for boys to play programming games, and was surprised when girls actively participated in such STEM-related games. (Amber)

Amber was unconsciously influenced by gender stereotypes although she believed that boys and girls should be treated equally. Such a conflict reinforces Stephenson et al.’s (2021b) observation of the gap between teachers’ verbal statement of gender equity and genuine perceptions.

Gullberg et al. (2017) indicate, although some ECEs’ biased beliefs seem innocent and imperceptible, ignoring these subtle biases in the long term could continually internalise girls’ self-perceptions and identities. Therefore, eliminating ECEs’ potential gender biases becomes crucial to avoid the persistent suppression on girls’ STEM development.

#### Theme 3: Varied Purposes and Focuses of Engagement in Boys’ and Girls’ STEM Play

Although the participants acknowledged boys’ and girls’ similar interests in STEM, they felt that different genders seem to bear different purposes and disparate focuses when utilizing STEM-related skills. For example, Emily shared a photograph to demonstrate how children in her class engaged in *TinkerToy* (an engineering toy with a set of wooden dowels, joints and wheels):

You can find [in this photo] that boys and girls were creating artifacts with wooden dowels. When I asked them what they were creating, boys were motivated by “how tall the building was” or “how long the guns were”, while girls preferred to endow these materials with some virtual meaning, such as, “I am making a fairy wand to cast spells.” (Emily)

Rennie added that boys and girls tend to have distinctly different themes in construction games:

Boys like building space stations, guns, and spacecrafts. They also tend to modify and interact with their artefacts after the construction. Girls, on the other hand, like building artefacts based on role play themes following certain plots. New social plays, such as inviting small animals [toys] to their homes, are often created after they finish building. (Rennie)

Sarah believed girls and boys show different levels of complexity and depth of using STEM knowledge. In her viewpoint, many girls exceed boys in creative and divergent thinking in STEM play, as girls can create innovative meanings for play by integrating their acquired knowledge. She noted:

In construction games, boys are usually keen on assembling limited shapes, such as guns and hammers, while girls are often more imaginative. For instance, many girls tried to apply their freshly acquired mathematic knowledge to create different geometric shapes. I was very surprised by their awareness of interdisciplinary activities in STEM games. (Sarah)

These examples demonstrate ECEs’ perceptions that boys and girls endow different meanings to STEM play with a similar level of interest and engagement. Boys might be pragmatically oriented, while girls are intrigued by the characters’ social roles. Such a standpoint aligns with the findings of Gold (2014) and Hallström et al. (2014), who assert that boys regard the process of constructing and building as the centre of their play, whereas girls prefer to utilize technological skills as an aid to achieve their social purposes. On the other hand, Sarah’s belief that girls’ creativity exceeds that of boys opposes the finding of Coyle and Liben (2018), who acknowledge boys’ higher STEM-related skills with the capability of independent thinking, and girls’ tendency to follow steps of instructions in STEM games. However, Sarah’s claim of girls’ outstanding mathematical skills seems to demonstrate the invisible gender gap in Chinese students’ mathematics achievements (OECD, 2015; Tsui, 2007). Such a paradox between girls’ mathematical performance and STEM aspirations could generate new debates and reflections.

Overall, these findings offer a glimpse of Chinese ECEs’ conscious attention to the different performances and preferences of boys and girls in their STEM development, which could assist educators in developing their targeted instruction to individual children or groups. Meanwhile, the participants’ narratives also suggest their courage to confront the stereotypical discourse of females’ incompetence in STEM, enabling girls’ voices and achievements in STEM-related regions to be noticed (Sarseke, 2017).

### What do Chinese ECEs See as Obstacles to Girls’ Engagement in STEM Play?

#### Theme 4: Traditional Perceptions From the External Environment Shape Lower Expectations of STEM for Girls

All participants considered the foremost challenge in promoting gender-inclusive STEM play is the traditional perceptions from the external environment, which inadvertently rationalise the disadvantaged position of girls in STEM. In this study, environmental impacts are found at three levels: culture and society, media, and family.

The Chinese cultural heritage of “male superiority” continuously shapes gender roles in contemporary society. As Confucian culture advocates women’s domesticity (Yang & Gao, 2019), societal expectations deem girls unsuitable for jobs that require high intelligence, such as STEM-related careers. This patriarchal ideology could influence children’s development in EC settings, as May indicated: “The stereotypical discourse inherited from older generations progressively shapes girls’ beliefs that they are not as competent as boys in STEM.”

Furthermore, mass media are creating gender stereotypes that diminish girls’ STEM interests. Sarah and Rennie noted that children could establish their gender identities by imitating cartoon characters at an early age. These cartoons, however, may intentionally or unintentionally convey ideals of gender stereotypes. Sarah resignedly commented: “I used to play *Thomas and Friends* for children to suggest that females can also work in seemingly male-dominated industries. Unfortunately, very few cartoons with atypical female roles are available to children.” Sarah’s narrative corresponds to Okanda and Taniguchi’s (2021) finding that characters associated with STEM identities in Japanese cartoons are almost always male. Therefore, the gender bias presented in mass media seems to have filtered all the way down to children’s STEM play in their early years.

Moreover, conventional gender perceptions from families may constrain girls from pursuing activities in STEM fields. Cathy and Amber both indicated that many parents would enrol their daughters in artistic rather than STEM-related after-class activities. Emily, Sarah and Rennie also recognised that parents with ingrained gender views would focus on cultivating their daughters’ femineity, submitting to an idealised female role assigned by society. Parents, as children’s first teachers whose beliefs would transmit and significantly influence their children (Lin et al., 2018), are therefore partly responsible for their daughters’ lack of motivation in STEM play.

The interviews reveal that stereotypical gender patterns still implicitly and constantly perpetuate in children’s learning environment (Yang & Gao, 2019). From the cultural-historical perspective, these traditional gender notions, from a broader societal level or specific family unit, continuously interact with girls in constructing their STEM identity (Hedegaard, 2009; Ødegaard & Krüger, 2012). The stereotypical discourses that underestimate girls’ STEM-related capabilities could be infused and reinforced as they come of age, driving them to conform to and reproduce conventional societal expectations on women.

#### Theme 5: Gender-Biased Group Dynamics Dominate Girls’ Personal Interest in STEM

Peer influence in the EC environment was believed to be another key factor hindering girls’ engagement in STEM play. Emily and Rennie both indicated that children with the same gender tend to play together:

A boy or a girl rarely interacts and participates in play groups of the other gender alone, unless he/she has strong play preferences or special skills. For example, when a girl notices that boys quickly occupy the STEM area, she tends to find other available play resources with her peers [other girls], even though she is interested in the STEM area. (Rennie)

As girls are more inclined to play with each other (Børve & Børve, 2017), they might abandon their interests and choose their comfort zone of “girls’ group”. In line with Meland and Kaltvedt’s (2019) research that children may feel anxious about crossing traditional gender boundaries, the participants perceive girls as more likely to withdraw from STEM play under the pressure of group dynamics, thus gradually losing interest in STEM areas.

In addition, boys may have dominance over certain STEM areas (Fleer, 2019). Sarah stated: “Boys tend to occupy games related to constructing vehicles and weapons, which could discourage girls from participating”. Such a power tendency might stem from deep-rooted patriarchal structures that endow males potential privileged positions (Pardhan & Pelletier, 2017), thus explaining girls’ reluctance to challenge the dominance of STEM areas.

In this light, exploring and understanding potential barriers for girls’ STEM engagement, including external stereotypes and the power negotiation between boys and girls, might provide ECEs with opportunities to develop more appropriate practices for children’s STEM inclusion. This is critical to the rejection of unbalanced power relations with a feminist perspective (Hussénius et al., 2013).

### What are Chinese ECEs’ Practices to Empower Girls in STEM Play?

#### Theme 6: Break Down the Gender Dichotomy in Educational Practices

ECEs attempted to tear down the gender dichotomy by embedding STEM elements in girls’ frequent play areas. Emily upgraded girls’ favourite role play area by adding more STEM characters.

The girls are obsessed with role play games, so I prepared some new costumes for STEM characters, such as scientists, engineers and architects. The girls were clamouring for these new STEM roles in their role play area. (Emily)

Similarly, Sarah incorporated STEM subjects in floristry, an activity considered feminine:

I intentionally integrated STEM knowledge into my weekly floral activities to develop children’s multi-disciplinary skills. For example, counting flowers and cutting leaves into specified geometric shapes could hone children’s mathematic skills; learning the structure of plants and caring for them could enhance their scientific literacy. This strategy allowed the girls to show interest in science and maths, and involved boys in art and aesthetic creation. (Sarah)

Emily and Sarah’s inclusive practices helped foster gender-neutral play environments and encourage children to participate in “atypical” roles (Chapman, 2016). They re-examined the “girlish” games and neutralised the inherent gender dichotomy by innovatively integrating STEM elements. With the support of feminist discourses, these “atypical games” that challenge pre-established gender roles and boundaries could dismantle the perceived inequalities in girls’ STEM development and to free their repressed voices (Hussénius et al., 2013).

#### Theme 7: Collective Games to Defy Gender-Biased Learning Environment

ECEs attempted to improve girls’ STEM engagement by cultivating a gender-neutral learning environment in collective games. All participants affirmed that free play is valuable in providing children equitable opportunities to participate in STEM activities (Rushton & King, 2020). Amber prepared “a full range of play materials, to stimulate children’s potential STEM skills, and encourage them to make free choices”. Catherine tried not to “overly intervene or label activities for boys and girls in free play”, believing “all activities are driven by children’s personal interest, regardless of gender”. Overlapping with the Essentia discourse introduced in Swedish kindergartens, Emily and Catherine’s practices suggest that ECEs could diminish their role as the dominant player and grant children sufficient freedom to explore their own intentions (Gullberg et al., 2017).

On the other hand, Sarah suggested collective games with specific themes could motivate all children’s interests and competence in STEM:

I often design some scenario-based imaginary play by incorporating history or current events to develop children’s multidisciplinary skills. An example is “Mine Warfare” (a tactic used by the Chinese army in WWII). I organized the children to play soldiers and utilize prepared construction tools to make “mines”. All children were equally engaged in the task, completely silencing the gender discourse associated with STEM. (Sarah)

Corresponding to the research of Gold et al. (2015) and Stephenson et al., (2021a, 2021b), Sarah created a *PlayWorld* without explicit gender connotations by simulating a social scenario from reality. As children would reshape their social behaviours when interacting in imaginary spaces (Vygotsky, 1967), Sarah’s strategy suggests a valuable way to help girls challenge gender-biased discourses and re-establish perceptions of STEM identity.

While free play emphasises the freedom of choices based on children’s interests, imaginary play allows children to displace gender norms in STEM through collaboratively immersing in gender-neutral imaginative situations. Both strategies promise to improve the insufficient engagement of girls in STEM activities at various stages of children’s development in more targeted ways. Educators are thus empowered to create a genuinely play-based learning environment free from the gender-stereotypical discourse.

#### Theme 8: Educators’ Multiple Roles in Promoting Gender Equity in STEM Play

More in-depth discussion found that ECEs play multiple roles in improving girls’ participation in STEM play. All participants emphasised the importance of permeating in various aspects of children’s play processes and subconsciously influencing children’s motivation and behavioural patterns.

The participants valued their role as the facilitator who challenges all children’s current abilities. They raised targeted questions during the playtime, rather than “underestimate girls’ STEM abilities by only questioning boys based on preconceptions” as Sarah described. Acknowledging children’s *Zones of Proximal Development* enables each child’s voice to be heard (Vygotsky, 1998), especially for girls who have been neglected in STEM.

As facilitators, ECEs are aware of children’s playing preferences and encourage them to participate in different play areas to expand their interests and skills. Emily stated: “If I find that the girls are constantly occupied in the home corner area, I will encourage them to try some other games or swap the play content with boys”. Such sensible instructions during children’s play could support all children, regardless of gender, to equally engage and develop interests from play resources (Lynch, 2015).

Moreover, female ECEs found hands-on demonstrations in STEM fields could be more effective than verbal instructions to girls. Emily commented:

My perceived “tomboy” personality makes me less like a “typical female educator”. For instance, I usually repair broken appliances and furniture by myself and demonstrate the whole process in front of all children in my class. (Emily)

May recounted a similar experience:

I enjoy scientific inquiry activities myself, so I would regularly invite children to participate in developing and conducting such activities. When I involve in their play as a role model, children become more interested and concentrated. (May)

Children’s STEM knowledge is cultivated by imitation of everyday life (Bozhovich, 2004). Thus, Emily and May’s atypical gender roles and behaviours could be replicated by children, especially girls who share the same gender identity. In line with Master and Meltzoff’s (2017, p. 228) research, when girls find “like me” role models who actively engage in STEM activities, they may develop the idea that STEM activities are gender neutral.

In addition, ECEs should play the role of encouragers to increase girls’ STEM engagement. For instance, to attract girls’ attention to STEM fields, Catherine constantly praises them, often accompanied by exaggerated body language and facial expressions.

Rennie offers her encouragement through her company:

I usually spend 30-40 minutes with a group of children in STEM play, no talking but just observing. They often become more active and concentrated on their tasks with my company. Meanwhile, the quality of their STEM works noticeably improves. (Rennie)

The experiences from Catherine and Rennie indicate that ECEs’ encouragement and companionship allow children to feel more valued and supported when engaged in STEM activities, thus diminishing gender limitations imposed on them.

Overall, ECEs can play variable and overlapping roles, based on children’s varied STEM capabilities. This finding challenges the certainty and homogeneity of identity development, corresponding to the dynamic trait advocated by cultural-historical theory (John-Steiner, 1999).

## Conclusion

This research adopts a multiple-case study approach to explore six Chinese ECEs’ personal beliefs and practices regarding gender differences in STEM play to answer the three research questions: (1) What are Chinese ECEs’ perceptions about gender differences in STEM play? (2) What do Chinese ECEs see as obstacles to girls’ engagement in STEM play? (3) What are Chinese ECEs’ practices to empower girls in STEM play? The findings reveal that participants acknowledged and valued children’s equal participation in STEM play, but failed to preclude deep-rooted gender stereotypes, leading to contradictory ideas and practices. Meanwhile, the Chinese ECEs regarded stereotypes from the external environment and the dominance of peer influence as main barriers to gender inclusion in STEM play. Moreover, this research discusses various inclusive practices and emphasises the multiple roles of ECEs in promoting gender-neutral environments for STEM play.

### Limitations and Future Research

The data source of this research is relatively narrow. First, the small sample size of only six participants could hardly represent all Chinese ECEs’ general perceptions. As findings are obtained from participants’ unique perspectives, experiences and backgrounds, no inductive conclusions could be generalised (Kervin et al., 2016). Therefore, increasing the sample size appropriately could be a way to address this limitation in future research. A quantitative survey based on the interviewed questions can be designed and distributed to larger numbers of ECEs. Complementing qualitative information with quantitative data will achieve a powerful combination of breadth and depth, thus more credible and inducible conclusions (Fetters & Freshwater, 2015). Moreover, the qualitative data source was limited to interviews due to COVID-19 restrictions in China. Although interviewing techniques were applied to capture ECEs’ thoughts as accurately as possible, discrepancies such as subjective assumptions may still exist. Therefore, future research should aim to diversify data collection methods since ECEs’ insights can be obtained beyond what they say. Data may also be enriched by field observations on ECEs’ teaching practices and their real-life interactions with children. Such triangulation through comparing and contrasting multiple data sources can verify the participants’ answers in the interviews (Kervin et al., 2016), thereby increasing the reliability and accuracy of research findings.

Due to the dominance of female educators in the Chinese early childhood education sector, all six participants in this study were female, which may potentially restrict the generalizability of the findings. However, as Mies (1981) proudly advocates, such “conscious partiality” (p. 68) to explore the world from a feminist perspective is worth celebrating instead of being criticised. As a feminist study, this research conducted by female researchers and participated by female ECEs, offers an opportunity on exploring gender issues in early childhood from a female perspective. With the researcher being female too, such ‘woman-to-woman’ interviews are likely to establish a harmonious and supportive rapport that facilitates the sharing of personal experiences and feelings on gender issues (Archer, 2002).

Despite these limitations, this study is undoubtedly valuable since the aim of this study is not to draw definitive conclusions on the given topic, but rather to put forth a “prologue” that inspires further studies on this innovative domain of early childhood education in China.

### Implications

Previous studies indicate the significant gender differences in STEM achievements in developing countries, which could mainly stem from gender-labelled interests and motivations in children (Smith et al., 2015). Therefore, this research is significant for the Chinese EC sector, to fill the current research gap of lacking studies examining gender differences in STEM play in Chinese EC settings.

From ECEs’ perspectives, this research examines their perceptions and practices regarding potential gender stereotyping in children’s STEM play from a feminist perspective. ECEs could recognise factors that hinder gender equity, and re-evaluate their roles in shaping and influencing children’s gender identities. This study provides discussion of more inclusive strategies and professional practices, which subsequently support children’s engagement in STEM play in a non-biased environment.

For the Chinese ECE sectors, this research initiates an otherwise often ignored debate on the gender issues in STEM. Despite the small number of participants, the interviewed ECEs come from all over China with different teaching experiences. Such maximum variation sampling is intended to provide inspiration to educators and educational institutions with similar backgrounds and experiences (Bold, 2012).

Through the lens of cultural-historical theory, this study inspires ECE leaders to actively reform pedagogical policies, modify curriculum and enhance staff training. The findings provide support for them to continuously examine barriers to children’s STEM development in their educational environment and endeavour to eliminate potential inequities.

In summary, the researcher believes that educators, especially ECEs, are obliged to remain sensitive to such inequities. Besides valuing the significant influence of educators on children’s development based on the cultural-historical theory, the supplementary feminist discourse is crucial in this research since my intention is to advocate for girls’ equal competence and ultimately diminish gender stereotypes in EC settings. It is the researcher’s hope that this research focus may bear far-reaching implications for Chinese ECEs, young children (especially girls), and even the Chinese EC system.

## Data Availability

Due to the sensitive nature of our data, the data set will be not publicly shared.
